# Graph Machine Learning for Improved Imputation of Missing Tropospheric Ozone Data

**DOI:** 10.1021/acs.est.3c05104

**Published:** 2023-09-04

**Authors:** Clara Betancourt, Cathy W. Y. Li, Felix Kleinert, Martin G. Schultz

**Affiliations:** † Jülich Supercomputing Centre, Forschungszentrum Jülich, 52425 Jülich, Germany; ‡ Max-Planck-Institut für Meteorologie, 20146 Hamburg, Germany

**Keywords:** graph signal processing, graph machine learning, missing data imputation, air quality, tropospheric ozone

## Abstract

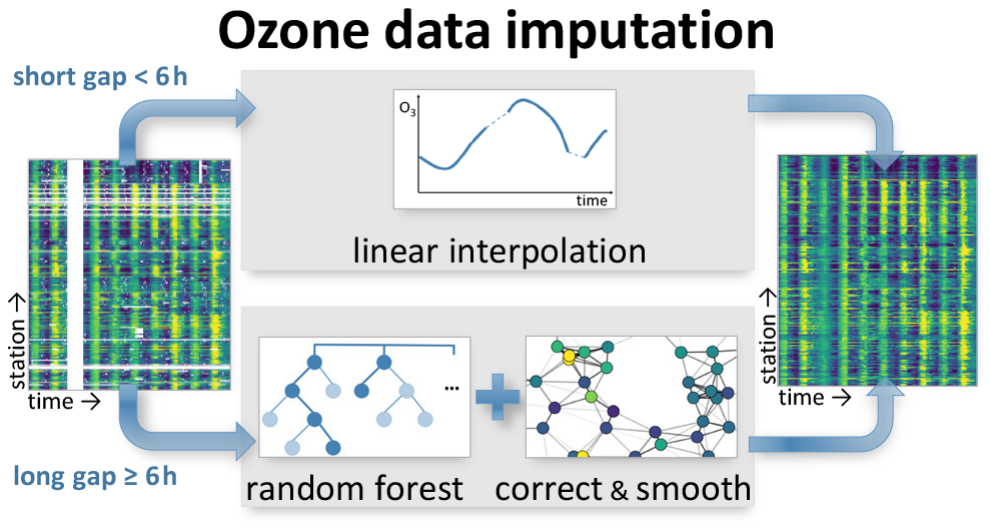

Gaps in the measurement series of atmospheric pollutants can impede the reliable assessment of their impacts and trends. We propose a new method for missing data imputation of the air pollutant tropospheric ozone by using the graph machine learning algorithm “correct and smooth”. This algorithm uses auxiliary data that characterize the measurement location and, in addition, ozone observations at neighboring sites to improve the imputations of simple statistical and machine learning models. We apply our method to data from 278 stations of the year 2011 of the German Environment Agency (Umweltbundesamt – UBA) monitoring network. The preliminary version of these data exhibits three gap patterns: shorter gaps in the range of hours, longer gaps of up to several months in length, and gaps occurring at multiple stations at once. For short gaps of up to 5 h, linear interpolation is most accurate. Longer gaps at single stations are most effectively imputed by a random forest in connection with the correct and smooth. For longer gaps at multiple stations, the correct and smooth algorithm improved the random forest despite a lack of data in the neighborhood of the missing values. We therefore suggest a hybrid of linear interpolation and graph machine learning for the imputation of tropospheric ozone time series.

## Abstract 2

Gaps in the measurements of atmospheric pollutants impede the assessment of their impacts and trends. We propose a new graph machine learning method for missing data imputation of the air pollutant tropospheric ozone.

## Introduction

Tropospheric ozone is a toxic air pollutant and a short-lived climate forcer.^1,2^ While stratospheric ozone protects life on earth from harmful ultraviolet radiation, tropospheric ozone is a health hazard^3−5^ and a substantial threat to global food security through the destruction of crops.^6−8^ Its surplus radiative forcing is estimated to be 0.39 W m^–2^, which is about a quarter of the radiative forcing of carbon dioxide.^9,10^ As a secondary air pollutant, ozone is formed by a cascade of (photo-)chemical processes in the atmosphere, which include precursors such as nitrogen oxides (NO_*x*_) and volatile organic compounds (VOCs).^11,12^ The interplay of chemistry, transport, and deposition induces daily and seasonal cycles in the distribution of ozone concentrations, which are superimposed with variances from all spatiotemporal scales.^1,12,13^ As such, ozone concentrations can change substantially in a matter of hours and on spatial scales of kilometers. It is therefore difficult to quantify the regional distribution of tropospheric ozone, and a fine-resolution monitoring network is required to obtain reasonably precise estimates of this distribution.^14,15^ The measurements, which are typically reported as hourly averages, are used to determine whether thresholds of ozone statistics (or ozone “metrics”) are exceeded^13,14,16^ and hence to assess the impacts of ozone at various times and locations.

Like any air quality monitoring time series, ozone measurements suffer from missing data. These can occur due to sensor malfunctioning, calibration procedures, issues with data transfer, or the stations going out of operation. Missing data reduces the robustness of statistical analyses.^13,17^ For example, if an ozone metric counts concentration threshold exceedances on a yearly basis and a sensor fails on a day with an exceedance, then a yearly statistic can be corrupted. Missing data also impede the usefulness of such data in other contexts. For example, machine learning models to forecast ozone concentrations^18−21^ require a gap-free time series as input to make predictions. It is therefore necessary to impute the gaps in the ozone concentrations.

Ozone metrics for air quality assessments are usually aggregated hourly measurements of longer time periods, e.g., one year. Often, missing data within the aggregation period are compensated by imputing the average concentration over this period for each missing value.^13,22^ This approach is often applied implicitly when an ozone metric is calculated on the basis of the available fraction of the data set. In order to ensure a certain level of robustness of the metric, this simple imputation method is generally only applied to time series with a maximum fraction of missing values of 25% or less.^14,22^ More advanced missing data imputation techniques for missing air pollutant data were developed during the past years.^23,24^ Univariate interpolation methods, e.g., linear interpolation and spline interpolation, depend on the available data at time steps before and after a gap and are therefore suitable for shorter gaps in the range of hours. In contrast, multivariate methods, which include linear regression and machine learning algorithms such as neural network and random forest, make use of auxiliary data or covariates such as meteorological data and are therefore suitable for longer gaps. The imputation performance depends not only on the amount of missing data but also on the manner in which data are missing, i.e., the “missingness”. Missing data patterns can be classified into three types according to the dependency of the missingness on the variable of interest and the auxiliary data:^25−27^ (1) missing completely at random (MCAR), where the data missingness is independent of the variable of interest and any other external influences; (2) missing at random (MAR), where the data missingness is independent of the variable of interest but the missing data pattern can be related to auxiliary data; and (3) not missing at random (NMAR), where the data missingness depends on the variable of interest. The missing data patterns in air quality monitoring are generally MCAR or MAR.^23,25,28^ In that case, the reasons why the data are missing can be ignored in the analysis of the data, and hence the methods used for missing data imputation can be simplified.^27^

Univariate and multivariate methods, or combinations of them, were successfully applied for missing air quality data imputation.^23,25,29,30^ However, even sophisticated machine learning methods fail to efficiently utilize available data at monitoring stations in the neighborhood of a missing measurement. Challenges in using these data arise because stations are irregularly placed and neighboring measurements may not be available for all time steps. One simple approach to include neighboring data to predict or impute air quality data is to consider spatial distances or correlations between the stations.^31−33^ A more advanced solution to this is graph machine learning,^34,35^ a subfield of graph signal processing^36,37^ which allows machine learning on irregularly structured data such as a monitoring network. Graph-based methods have been adopted for air quality-related tasks, such as outlier detection, postprocessing of low-cost sensor data, or high-resolution forecasting.^38−44^ Graph machine learning was shown to be suitable for the imputation of different data sets,^45−47^ yet, to the best of our knowledge, they have not yet been used to impute missing air quality data.

**Figure 1:**
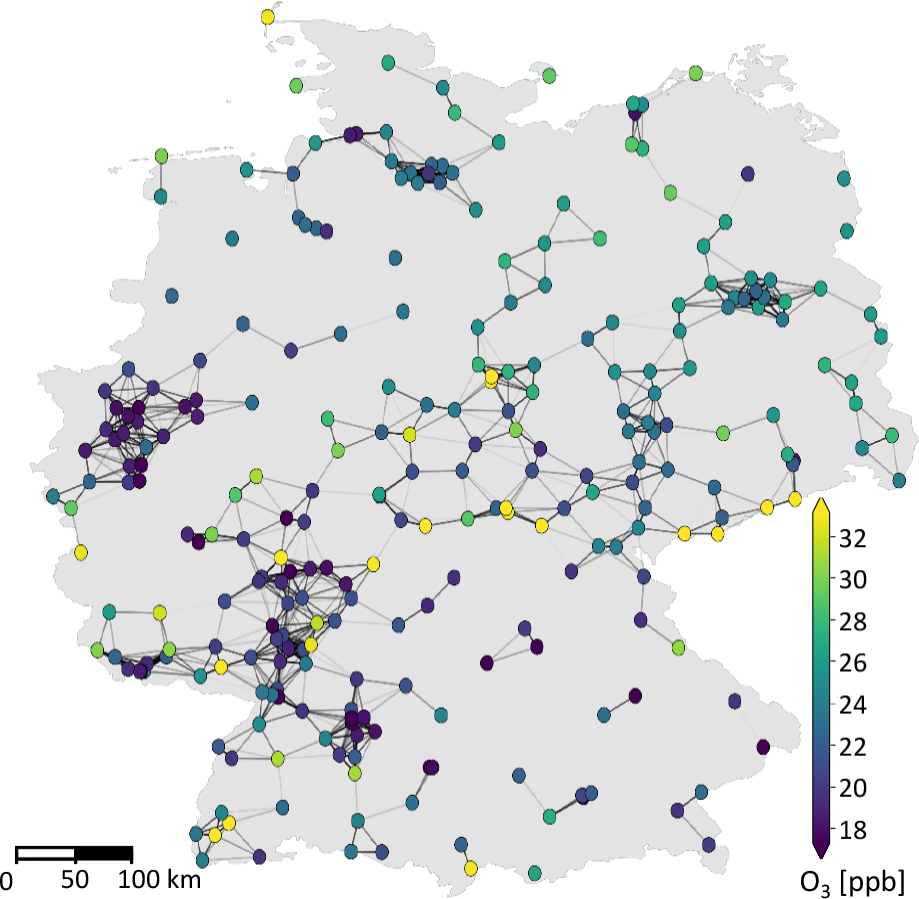
Illustration of the graph structure defined on the stations of the UBA monitoring network. The stations are nodes in the graph. Nodes of 50 km distance or less are connected by edges, which allow a graph machine learning algorithm to pass messages between them. In this figure, the nodes are labeled with the average ozone concentration over 2011, omitting temporal variances for clarity.

In this study, we develop a strategy to use graph machine learning to improve the imputations achieved by other existing methods. As a case study, we use a data set of hourly observations from 278 stations of the German Environment Agency (Umweltbundesamt – UBA) air quality monitoring network in the year 2011. Figure 1 shows the station locations and their relations in the graph that is built according to the procedures described in the next section. We combine the available observations with geospatial metadata, meteorological, and reanalysis data to allow the different regression and machine learning approaches to exploit relationships between these data and the measured ozone time series. For the analysis of the performance of these approaches, we identify three types of gaps that frequently occur: (1) shorter isolated gaps in the range of hours, e.g., when an instrument is offline for 1 h during calibration; (2) longer gaps in the range of months including multiple daily cycles and even changes in seasons; (3) gaps occurring at all stations of the network at the same time. The assessment of the three types of gaps suggests optimal imputation strategies for each gap type. We compare the performance of our method with published baseline statistical, numerical, and machine learning methods. Besides the code and input data, we also provide the final imputed version of the data set.

## Data and Methods

### Ozone Data

Ozone data used in this study are from the German Environment Agency (Umweltbundesamt – UBA). The UBA collects and provides air quality data for Germany. We extracted the data from the Tropospheric Ozone Assessment Report (TOAR) database^13^ at the Jülich Supercomputing Centre. The TOAR database receives a copy of all German ozone data from the UBA in near-real time. We selected hourly data from 278 stations across Germany in 2011 because there was an exceptionally large number of missing values in these data. It should be noted that UBA itself provides a final validated data set of ozone concentrations in the following year, which has fewer gaps than the data we have worked with. However, to develop our method and demonstrate its potential, we have chosen the preliminary data set with more frequent and larger gaps, and we use the final validated data set to crosscheck our results.

**Figure 2:**
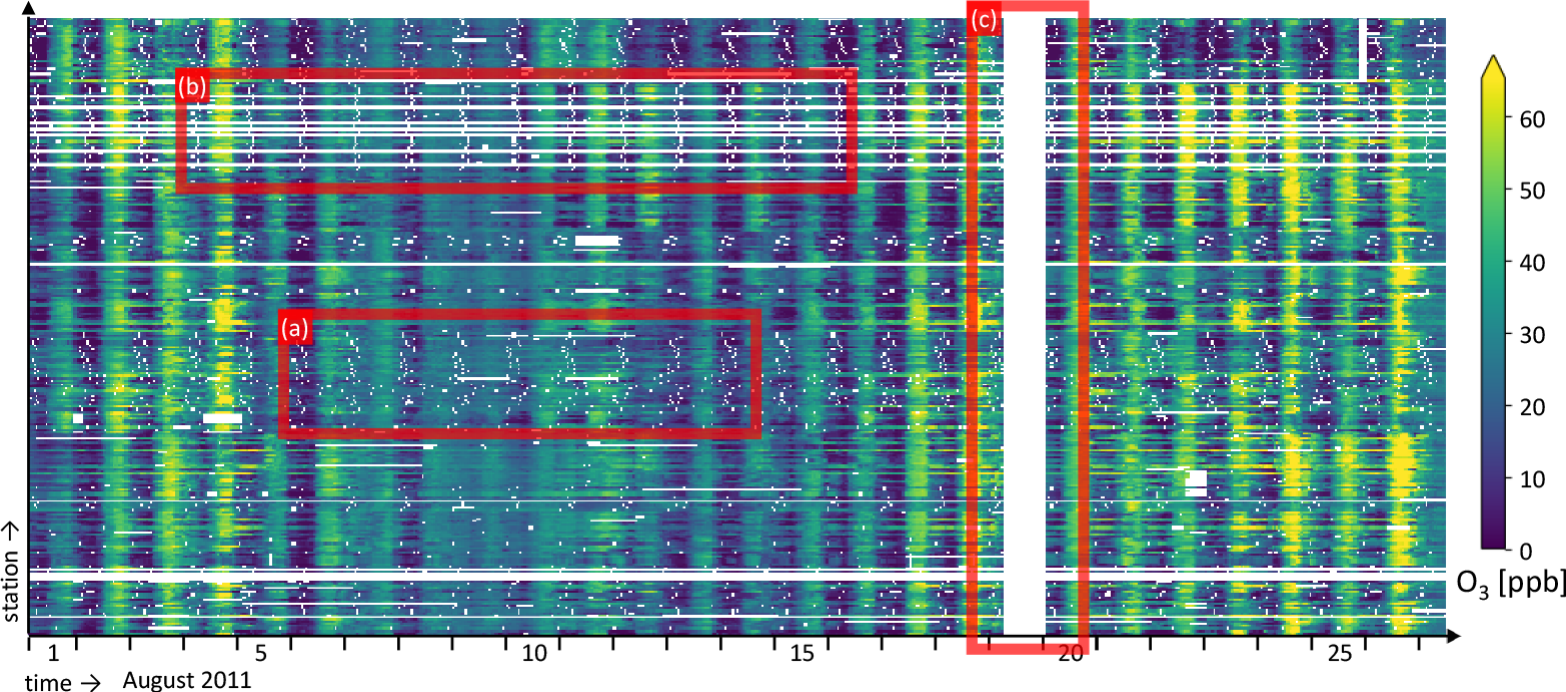
Measured ozone concentrations of August 1–26, 2011, at the selected UBA stations. Examples of three cases are marked: (a) short isolated gaps, (b) longer isolated gaps, and (c) gaps occurring at all stations simultaneously.

The selected data set contains over 2.4 million data points for training, testing, and evaluating the different imputation methods. The location of the stations and their mean ozone concentrations are shown in Figure 1. Compared to the theoretically available maximum number of hourly values, 15% of the data are missing. The missing data are generally completely random (MCAR), except for a few cases where sensors are offline during the night and thus missing randomly (MAR). Seventeen % of data gaps occur at single stations during short periods of up to 5 h length. 57% occur as longer periods at single stations, and 26% of the data gaps occur at all stations simultaneously. This last category contains several short gaps of 3–4 h and three longer gaps with 18–43 h length starting from August 19, October 8, and December 20, 2011, respectively. The latter gaps could be traced back to data transmission gaps between the UBA and the TOAR database and are not part of the original UBA data set. Figure 2 shows an excerpt of these data, including examples of the three missing data patterns. Section S1 of the Supporting Information contains the summary statistics of the data. A detailed overview of gap lengths is given in section S2.

### Auxiliary Data

We selected the following auxiliary data as features for multivariate imputation because they have been shown suitable to predict ozone in previous machine learning studies:^18,20,48^

- Datetime features: hour of the day, day of the week, and day of the year;
- Meteorological data: temperature, relative humidity, cloud cover, planetary boundary layer height, and wind components *u* and *v*;
- Atmospheric composition reanalysis data: concentrations of ozone (O_3_), nitrogen monoxide (NO), and nitrogen dioxide (NO_2_);
- Emission data: nitrogen oxides (NO_*x*_); and
- Static station metadata: altitude, relative altitude, population density, nightlight intensity, station type, and type of area.

Meteorological data were extracted from the 6 km hourly resolution COSMO reanalysis^49^ (COSMO-REA6). Atmospheric composition reanalysis data were extracted from the surface level of the ECMWF Atmospheric Composition Reanalysis 4 (EAC4) data set,^50^ which combines observations with model data from a global chemical transport model (CTM). The emission data were extracted from the CAMS Global anthropogenic emissions (version 5.3)^51^ with monthly resolution. Station metadata are taken from the TOAR database.^13^ Here the relative altitude is the difference in elevation to the lowest point 5 km around the station.

### Missing Data Imputation with Mean Values

As a statistical baseline method (*B*), we impute the spatiotemporal mean (*stm*) over all available data to all gaps:
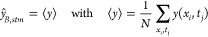
1*ŷ* denotes an imputed value, *y* is a measurement, *N* is the total number of available measurements, *x*_*i*_ is a station with index *i*, and *t*_*j*_ is a time step with index *j*. As a variant of this method, we impute the time-dependent spatial mean (*sm*), which is the mean over all available measurements at a specific time step:
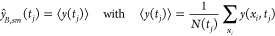
2

If no data are available for a given time step (which is the case for 352 of 8760 time steps), we impute the mean ozone concentration from EAC4 of that time step. This method captures daily, weekly, and seasonal cycles inherent in the available data without regard for extra station information or meteorology.

### Missing Data Imputation with the Nearest-Neighbor Hybrid Method

A second statistical baseline method is the hybrid of linear interpolation (*lin*) for short gaps and multivariate nearest-neighbor (*nn*) interpolation for longer gaps. This method has been shown to be effective for missing data imputation of air pollution data.^23^ The authors called it the nearest-neighbor hybrid (*nnh*), and we adopt their naming convention. According to this method, shorter gaps with a length *L* shorter than a threshold length *L*_*t*_ are imputed by fitting a straight line between the two end points *t*_1_ and *t*_2_ of the gap and calculating the missing values for any time *t*_1_ < *t*_*j*_ < *t*_2_ from this line equation. *L*_*t*_ is a tunable hyperparameter and varies according to the air pollutant in question. Longer gaps are imputed by multivariate nearest-neighbor interpolation as follows: The auxiliary data (features) of a data point are considered to be points f⃗ in the multidimensional feature space. In this study, we use 19 features, so this space has 19 dimensions. For every missing ozone value with index *k*, the nearest-neighbor sample with index *k*′ with an available ozone measurement is searched in the feature space. Thereby, all features are standardized to zero mean and unit variance, so features covering different scales are treated with equal importance. The distance measure is the Euclidean distance. Thus, in effect, the imputed ozone value of a missing data point is calculated according to eqs 3–5:
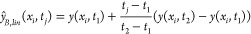
3
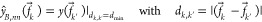
4
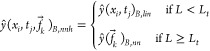
5

An arrow  above a variable denotes a vector in this and all following equations.

### Missing Data Imputation with Atmospheric Reanalysis

Imputation with ozone values from atmospheric reanalyses (here EAC4) is another baseline method against which machine learning models can be compared. To obtain the EAC4 reanalysis, observations from multiple satellites were assimilated with ECMWF’s Integrated Forecasting System^50^ (IFS). The model’s prior estimates are optimized through minimizing the cost function, which measures the difference between modeled and observed fields to produce an improved estimate over the reanalysis period. Without the time constraint of issuing timely forecasts, the quality of reanalysis products benefits from the improvement of the quality and availability of observations. The EAC4 data are available in gridded format with 80 km spatial resolution and 3 h temporal resolution. We impute the ozone concentration from the EAC4 data set of the nearest-neighbor grid cell to all gaps:
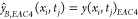
6

We point out that the imputation of measurements with gridded data is not ideal due to the representation mismatch of points and grid boxes. Furthermore, this method is prone to model biases that cannot be completely removed with statistical bias correction methods.

### Random Forest for Missing Data Imputation

Random forest is a tree-based machine learning algorithm developed by Breiman in 2001.^52^ Tree-based models were proven to excel in particular on tabular style data like the auxiliary data of this study.^53^ A random forest is an ensemble of decision trees for classification or (in our case) regression. Decision trees iteratively partition the training data by finding logical rules associated with the input features to minimize a cost function such as squared loss. Individual decision trees have a low bias but are prone to overfitting. A random forest improves this problem through the resampling of the available training data. It is obtained by fitting many, usually several hundred, decision trees on bootstrapped training data sets. We chose random forest because in preliminary experiments it outperformed other machine learning models. In particular, gradient boosted trees^54^ performed slightly worse than random forest on our data set, presumably because they are more prone to overfitting on noisy data with many variables such as ours. We rejected linear models because they failed to capture the ozone cycles and nonlinear relationships with the input features in our preliminary experiments.

In this study, a random forest (*rf*) is fitted on the features as inputs and the available measurements as output. The features are the auxiliary data introduced earlier. This random forest predicts an estimate of ozone concentration for every missing ozone measurement, based on the features of that data point:
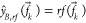
7

### Defining a Graph Structure on an Air Quality Monitoring Network

Graphs are a “general language for describing and analyzing entities with relations or interactions”.^55^ Machine learning on graphs has gained success in the past years because it can solve complex tasks on data of irregular structure, such as protein folding, traffic prediction, or action recognition in computer vision.^56−58^ From a graph theoretical perspective, the task in this study is to provide labels for unlabeled nodes (in our case, data points with missing ozone values).

We define the graph structure of our data in the following way: Each data point at station *x* and time step *t* is a node; therefore, there are ca. 2.4 million nodes in total. If there is a measurement *y* available for that data point, then the node is labeled with that measurement. If not, it is unlabeled. So in our case, 15% of the nodes are unlabeled. Every node has features f⃗, namely, the 19 auxiliary data values described above. An edge exists between the nodes *k* and *k*′ if two conditions are fulfilled: first, they are 50 km or closer in spatial distance, and second, the time difference between them is 6 h or less. We chose these thresholds because the areas of influence of two measuring stations overlap at a distance of 50 km or less^14^ and because ozone varies on hourly scales. The edge allows node *k*′ to receive information from node *k*. The total number of edges obtained in this way is about 240 million, so each node receives information from about 100 nodes on average. The edges are weighted according to the spatial and temporal distances Δ*x*, Δ*t*:
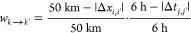
8

Figure 1 illustrates the graph, omitting the time component and self-loops for clarity. An isolated node in this figure has neighbors only in the temporal domain, so message passing will only be possible along the temporal axis. For more information on graph theory, the reader is referred, for example, to the book by Hamilton.^34^

### Graph Machine Learning To Improve Missing Data Imputation

Graphs are routinely used in semisupervised missing data imputation, where information from both labeled and unlabeled data are used.^59−61^ In particular, the correct and smooth algorithm by Huang et al. has proven effective in such tasks.^60^ Correct and smooth is a graph machine learning method, since there is an iterative improvement of predictions based on message passing within the graph. It is about 100 times faster to fit than a graph neural network.^58,60^

We apply this algorithm to improve the baseline imputation methods described above. As the algorithm was originally designed to output class probabilities in semisupervised classification tasks, we had to make minor adjustments to apply it to the imputation of ozone concentrations, which is a regression task. The original method predicts a label score for every class and then converts all label scores to class probabilities by applying a softmax function. We modified the method to have only one output as we impute only one variable (ozone). We also removed the softmax function, which is unnecessary for regression problems. To the best of our knowledge, this is the first study in which the correct and smooth algorithm is used for a regression task rather than a classification task.

The correct and smooth algorithm is applied in three steps. In the first step (“estimate”), a base model *B* estimates the node labels *ŷ* based on the node features without making use of the graph structure. In this study, the base model is any of the statistical or machine learning methods described above:
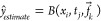
9

In the second step (“correct”), the errors *e*_*k*_^0^ of this base model are calculated by comparing the predicted labels to the true labels wherever possible. These errors are then propagated iteratively *L*_1_ times to the unlabeled nodes, and the resulting error correction is added to the base prediction. This step, also called “residual propagation”, assumes that if nodes *k* and *k*′ are connected by an edge, their errors *e*_*k*_ and *e*_*k*_^′^ of the base model are correlated.
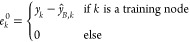
10
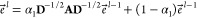
11
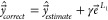
12

Here, **A** with *A*_*k*,*k*′_ = *w*_*k*→*k*′_ is the adjacency matrix of the graph that contains the scaled edge weights as entries. **D** is the degree matrix of the graph that contains the node degrees as diagonal entries; therefore, **D**^–1/2^**AD**^–1/2^ is the normalized adjacency matrix. The index *l* denotes an iteration step between 0 and *L*_1_. *L*_1_, α_1_ and γ are tunable hyperparameters.

The third step (“smooth”) is similar to the second step, but here the labels *y* and  are propagated because it is assumed that neighboring nodes have similar labels. This assumption is valid because ozone concentrations are correlated in space and time.^62^
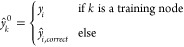
13
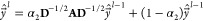
14
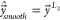
15

The smoothing step therefore resembles a graph filter.^63^*L*_2_ and α_2_ are tunable hyperparameters.

### Evaluation

To evaluate the different imputation methods, we must artificially mask a share of the labeled data points as missing and compare the imputed ozone concentrations to the originally reported values. In machine learning, it is common to reserve a large share of labeled data for fitting the models (“training set”) and smaller shares to tune hyperparameters (“validation set”) and to test the final model performance (“test set”). Therefore, we split the data as follows: 70% of the data are used as is for training. Fifteen % of the data are masked (i.e., labels are removed), and of these, half are assigned to the validation set and half to the test set. The remaining 15% of the data are unlabeled samples. These are the missing data samples described above. To realistically test the predictive performance of the different algorithms, we maintained the gap characteristics of the missing data in the masking of the validation and test sets. For every gap length found at single stations, we mask counterparts of equal length randomly in the validation and test sets. Similarly, we mask counterparts of the gaps occurring at multiple stations. See section S2 for a detailed list of gaps masked for validation and test purposes.

We used three evaluation metrics that are commonly used for missing data imputation. The coefficient of determination *R*^2^ is unitless and measures the proportion of variance in the true values that is explained by the model. A larger *R*^2^ denotes a better model, and the largest possible value is 1.
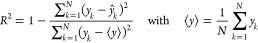
16

We also evaluate the root-mean-square error (RMSE) in ppb:
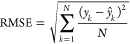
17

Obviously, perfect agreement would yield an RMSE of zero. The third evaluation metric is Willmott’s index of agreement,^64^ which measures the degree to which a model’s predictions are error-free. It can point out the total discrepancies between the imputations and the observations that are not captured by the index of agreement. It is unitless, and its largest possible value is 1.
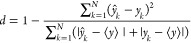
18In eqs 16–18, *k* denotes a sample index, *N* is the total number of samples,  is an imputed ozone value, and *y*_*k*_ is a measured ozone value.

To ensure robustness of the imputation methods and hyperparameters, we iteratively generate ten versions of the aforementioned data splits and compare their evaluation results. We also produce an imputed data set with the best method and hyperparameters and crosscheck this imputation with the final validated data set UBA provides.

## Results

This section is organized as follows: First, we describe hyperparameter tuning and model fitting. Then follows the evaluations of three distinct missing data cases: short gaps of up to 5 h length, longer gaps, and gaps at multiple stations. We then consolidate the findings for the three cases into a combined imputation. Lastly, we describe the production of the final imputed data set.

### Hyperparameter Tuning and Model Fitting

To tune the hyperparameters for the nearest-neighbor hybrid (*nnh*), the random forest (*rf*), and the correct and smooth postprocessing, the models were fit on the training set and evaluated on the validation set. The *nnh* model (eqs 3–5) has only the parameter *L*_*t*_. We tuned this parameter by starting with a threshold length of 1 h and increasing it in steps of 1 h. The best evaluation metrics were found for a threshold length of *L*_*t*_ = 6 h. For the random forest (*rf*, eq 7), 500 trees were initially trained with unlimited depths. To avoid overfitting, the maximum depth was then diminished, until the training and validation errors were the same. This resulted in a depth of 15. Features for the random forest were selected by forward feature selection.^65^ As a result, all features were selected except the reanalyzed NO concentration. The parameters α_1,2_, *L*_1,2_, and γ of correct and smooth (eqs 9–15) were tuned by grid search. Details and results of the hyperparameter tuning can be found in section S3.

To fit the final models, which are analyzed in the following, the optimal hyperparameters were used and models were fitted on both the training and validation sets. The models were then evaluated on the test set.

### Imputation of Short Gaps

Table 1 shows the evaluation metrics of the imputation results of short gaps up to a length of 5 h. The nearest-neighbor hybrid (*nnh*), which carries out a linear interpolation (*lin*) for these gaps, performs best. Its *R*^2^ values are between 0.91–0.97, RMSEs are between 2.43–4.44 ppb, and *d* is ≥0.98. This agrees with the results of Junninen et al.,^23^ who found linear interpolation to be most effective for short gaps. As expected, the performance of the linear interpolation drops with the length of the gap as this method does not consider auxiliary variables or the daily cycle of ozone concentrations.

**Table 1:** Evaluation Results for Short Gaps

gap length	model		*R*^2^	RMSE [ppb]	*d*
1 h	spatiotemporal mean		0.00	15.82	0.02
		+ correct and smooth	0.70	8.67	0.91
	spatial mean		0.63	9.60	0.88
		+ correct and smooth	0.82	6.62	0.95
	nearest-neighbor hybrid		**0.97**	**2.43**	**0.99**
		+ correct and smooth	0.96	3.07	0.99
	EAC4 reanalyses		0.53	10.84	0.86
		+ correct and smooth	0.81	6.91	0.94
	random forest		0.85	6.15	0.96
		+ correct and smooth	0.90	4.94	0.97
2 h	spatiotemporal mean		0.00	15.82	0.02
		+ correct and smooth	0.65	9.36	0.89
	spatial mean		0.61	9.92	0.87
		+ correct and smooth	0.79	7.17	0.94
	nearest-neighbor hybrid		**0.96**	**3.33**	**0.99**
		+ correct and smooth	0.94	3.91	0.98
	EAC4 reanalyses		0.50	11.22	0.85
		+ correct and smooth	0.78	7.50	0.93
	random forest		0.84	6.26	0.95
		+ correct and smooth	0.89	5.34	0.97
3–5 h	spatiotemporal mean		0.00	15.12	0.02
		+ correct and smooth	0.60	9.54	0.87
	spatial mean		0.64	9.55	0.87
		+ correct and smooth	0.76	7.27	0.93
	nearest-neighbor hybrid		**0.91**	**4.44**	**0.98**
		+ correct and smooth	0.90	4.82	0.97
	EAC4 reanalyses		0.49	10.84	0.85
		+ correct and smooth	0.74	7.72	0.92
	random forest		0.81	6.64	0.94
		+ correct and smooth	0.86	5.68	0.96

### Imputation of Longer Gaps

Table 2 shows the evaluation metrics of the imputation of gaps that are 6 h or longer. The random forest in connection with correct and smooth performs best for these gaps, with *R*^2^ values of 0.86–0.87, RMSEs of 5.64–6.18 ppb, and *d* = 0.96. Correct and smooth postprocessing decreased the RMSE of the random forest by 0.57–0.76 ppb. With *R*^2^ values of 0.69–0.75, the nearest-neighbor interpolation is a suitable statistical method for missing data imputation but is consistently outperformed by the random forest.

**Table 2:** Evaluation Results for Long Gaps

gap length	model		*R*^2^	RMSE [ppb]	*d*
6–23 h	spatiotemporal mean		0.00	15.78	0.00
		+ correct and smooth	0.55	10.54	0.84
	spatial mean		0.64	9.38	0.88
		+ correct and smooth	0.75	7.88	0.92
	nearest-neighbor hybrid		0.75	7.85	0.93
		+ correct and smooth	0.79	7.13	0.94
	EAC4 reanalyses		0.56	10.47	0.87
		+ correct and smooth	0.73	8.22	0.92
	random forest		0.84	6.34	0.95
		+ correct and smooth	**0.87**	**5.65**	**0.96**
1–6 days	spatiotemporal mean		0.00	14.93	0.03
		+ correct and smooth	0.52	10.32	0.82
	spatial mean		0.59	9.56	0.87
		+ correct and smooth	0.71	8.00	0.91
	nearest-neighbor hybrid		0.72	7.85	0.93
		+ correct and smooth	0.78	7.07	0.94
	EAC4 reanalyses		0.47	10.84	0.85
		+ correct and smooth	0.70	8.22	0.91
	random forest		0.81	6.40	0.95
		+ correct and smooth	**0.86**	**5.64**	**0.96**
≥7 days	spatiotemporal mean		0.00	16.25	0.01
		+ correct and smooth	0.57	10.62	0.84
	spatial mean		0.67	9.27	0.89
		+ correct and smooth	0.77	7.76	0.93
	nearest-neighbor hybrid		0.69	9.00	0.92
		+ correct and smooth	0.75	8.12	0.93
	EAC4 reanalyses		0.56	10.81	0.87
		+ correct and smooth	0.75	8.17	0.92
	random forest		0.83	6.75	0.95
		+ correct and smooth	**0.86**	**6.18**	**0.96**

Table 2 also shows how correct and smooth, which relies on available data at neighboring stations for long gaps, improves the base models. Its effectiveness shows best with base models of low complexity. One example is the spatiotemporal mean which imputes the same constant to all gaps. The *R*^2^ value of this method alone is zero, because there is no variance in the imputations. Correct and smooth postprocessing increased the *R*^2^ values of the spatiotemporal mean by 0.52–0.57. This improvement is achieved only by passing information from neighboring stations across the graph edges defined in the given monitoring network. Although the correct and smooth algorithm is iterative, information on the same station from distant time steps is not propagated into longer gaps because the autoscale option of the algorithm reduces the influence of training nodes on unlabeled nodes with the number of “hops”. We therefore neglect autocorrelation of ozone values for times longer than the diurnal cycle.

**Figure 3:**
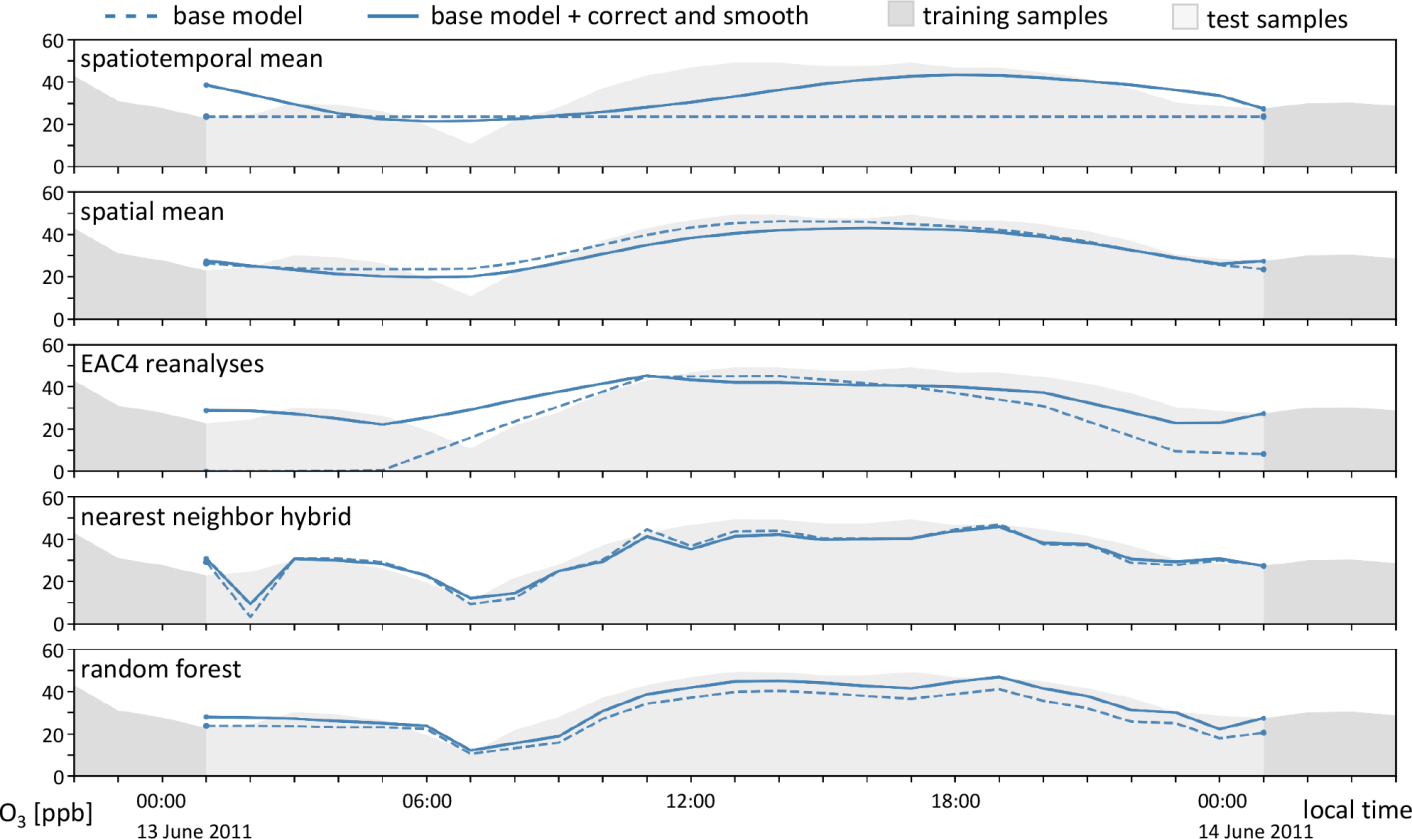
Example imputations of an isolated 24 h gap at station ‘DEHE001’ in the city of Darmstadt. The dashed lines are the imputations from the base models. The solid lines are the correct and smooth imputation postprocessed base models.

Figure 3 shows the imputed concentrations of the different methods using a 24 h gap at an urban background station in the city of Darmstadt (UBA id ‘DEHE001’, TOAR id 3443) as an example. There are 18 stations in the radius of 50 km around this station with distances of 11.8–49.9 km, and it can receive information from these stations across the defined graph edges. In the case of spatiotemporal mean, correct and smooth postprocessing could introduce a daily cycle. It also improved the other base models, even though they already predicted the daily cycle. The random forest has low errors but is improved slightly by being correct and smooth.

### Imputation of Gaps at Multiple Stations

Table 3 shows evaluation metrics of gaps occurring at all stations simultaneously. Similar to the gaps occurring at single stations, the nearest-neighbor hybrid (which carries out a linear interpolation for short gaps) reaches the best evaluation metrics for gaps of up to 5 h length. The longer gaps are still imputed best by the random forest in combination with correct and smooth, yet correct and smooth improved the RMSE by only 0.07 ppb in this situation. This can be explained by the fact that no neighboring data are available. Hence, the imputation has to rely on the features alone, which generally results in lower evaluation metrics.^48,66^

**Table 3:** Results for the Gaps at Multiple Stations^a^

gap length	model		*R*^2^	RMSE [ppb]	*d*
3–5 h	spatiotemporal mean		–0.01	15.39	0.11
		+ correct and smooth	0.63	9.32	0.89
	spatial mean		0.42	11.67	0.77
		+ correct and smooth	0.67	8.75	0.89
	nearest-neighbor hybrid		**0.92**	**4.45**	**0.98**
		+ correct and smooth	0.90	4.64	0.97
	EAC4 reanalyses		0.46	11.32	0.81
		+ correct and smooth	0.72	8.06	0.91
	random forest		0.78	7.06	0.93
		+ correct and smooth	0.84	5.95	0.96
1–6 days	spatiotemporal mean		–0.04	13.07	0.24
		+ correct and smooth	–0.13	13.64	0.31
	spatial mean		0.37	10.19	0.80
		+ correct and smooth	0.43	9.60	0.82
	nearest-neighbor hybrid		0.51	8.94	0.87
		+ correct and smooth	0.59	8.18	0.88
	EAC4 reanalyses		0.42	9.77	0.85
		+ correct and smooth	0.55	8.57	0.87
	random forest		0.78	5.95	0.93
		+ correct and smooth	**0.79**	**5.88**	**0.94**

a The nearest-neighbor hybrid method is a linear interpolation for 3–5 h gaps and a nearest-neighbor interpolation for longer gaps.

### Combined Imputation

**Figure 4:**
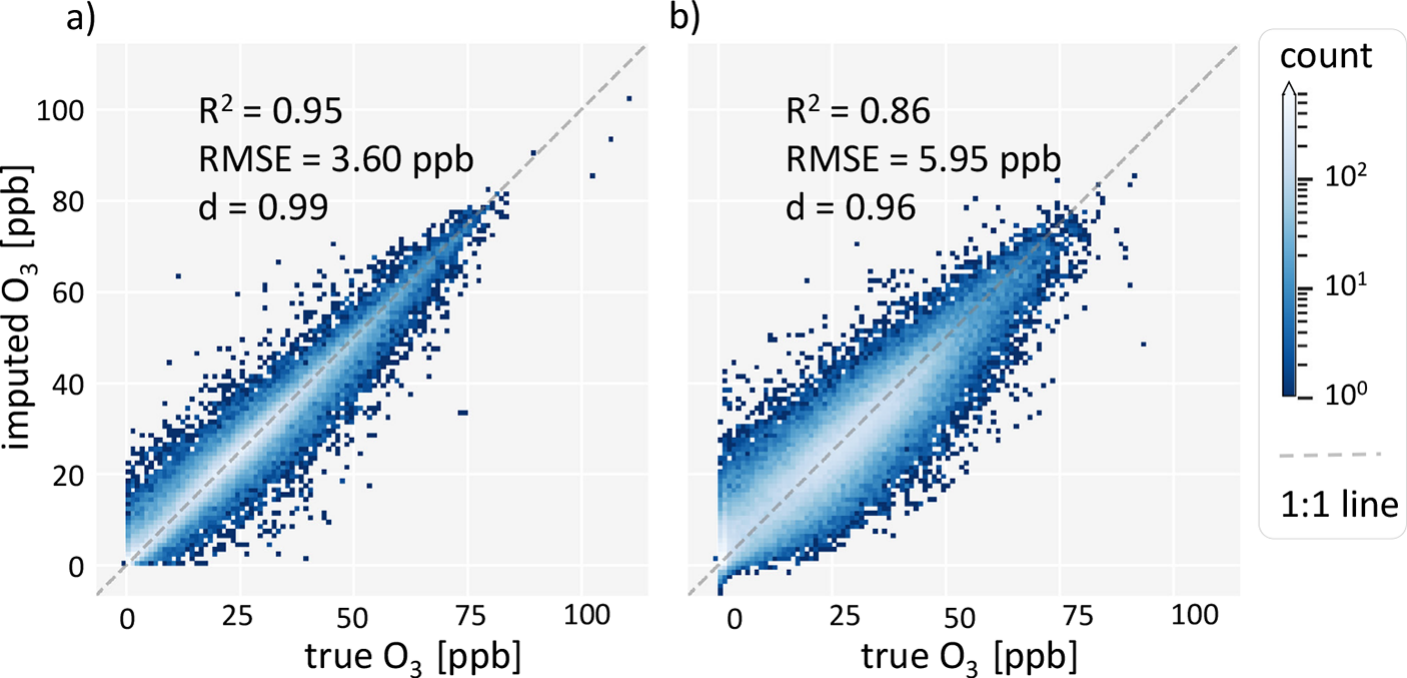
Heatmap of true versus imputed concentrations. (a) Short gaps of up to 5 h length, imputed by linear interpolation, and (b) random forest + correct and smooth for long gaps. This figure does not differentiate between isolated gaps and gaps at all stations.

According to the results presented in Tables 1–3, we created a combined imputation to evaluate our developed method. We imputed all short gaps with a length of up to 5 h with linear interpolation and all longer gaps with random forest and correct and smooth. We did not differentiate between gaps at a single station or at multiple stations since these methods are shown to be most effective, regardless of whether a gap occurs at one station or at multiple stations. The evaluation metrics of the complete test set and the iteratively generated data splits are shown in Table 4. They indicate the robustness of the imputation method. Figure 4 shows heatmaps of true and imputed concentrations, with differentiation between short and long gaps.

**Table 4:** Evaluation Metrics of the Test Set and Spread in Iterative Data Splits

evaluation metric	test set	ten iterative data splits
*R*^2^	0.89	0.89 ≤ *R*^2^ ≤ 0.90
RMSE [ppb]	5.13	5.52 ≥ RMSE ≥ 5.12
*d*	0.97	0.97 ≤ *d* ≤ 0.97

**Figure 5:**
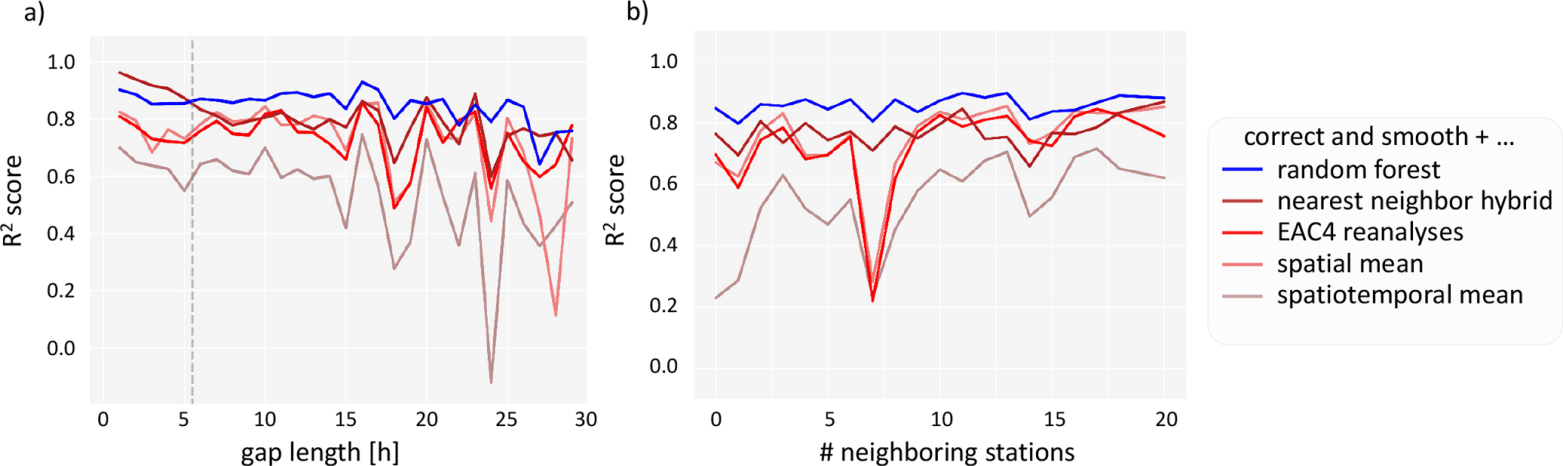
*R*^2^ evaluation metric vs gap characteristics. (a) Different gap lengths up to 30 h. The dashed line marks the gap length 5 h. This is when the final imputation model changes from linear interpolation to random forest + correct and smooth. (b) Number of neighbors in a radius of 50 km around the station. This plot only contains data from isolated gaps longer than 5 h.

Figure 5 shows a summary of how gap characteristics affect the evaluation metric *R*^2^ for the different base models in combination with correct and smooth. The *R*^2^ value generally decreases with an increasing gap length. Furthermore, there is a weak trend in improved *R*^2^ when more neighboring stations are available. Both trends are more apparent for the simple base models, such as the spatial mean and the spatiotemporal mean. The random forest in connection with correct and smooth, which has the best evaluation metrics, is also least affected by variations of the gap characteristics.

### Imputed Data Set

The imputed data set, which was produced within the scope of this study, is available under the DOI 10.23728/b2share.04821864a81f40af89c7633889f147cb. To produce this data set, we imputed all missing ozone data using the combined imputation and the trained random forest model. Note that data points that were masked for validation and testing were unmasked again in this final output data set; i.e., for these samples, the original measured ozone values are reported. About 180,000 samples that are missing in the preliminary UBA data set which we used to develop our method are present in the final data set which UBA provided in the following year. This is approximately 7.3% of the theoretically available samples. Cross-checking these with our imputations yields an *R*^2^ of 0.83, an RMSE of 5.63 ppb, and an index of agreement of 0.95. The evaluation metrics are slightly inferior to those reported in Table 4.

**Figure 6:**
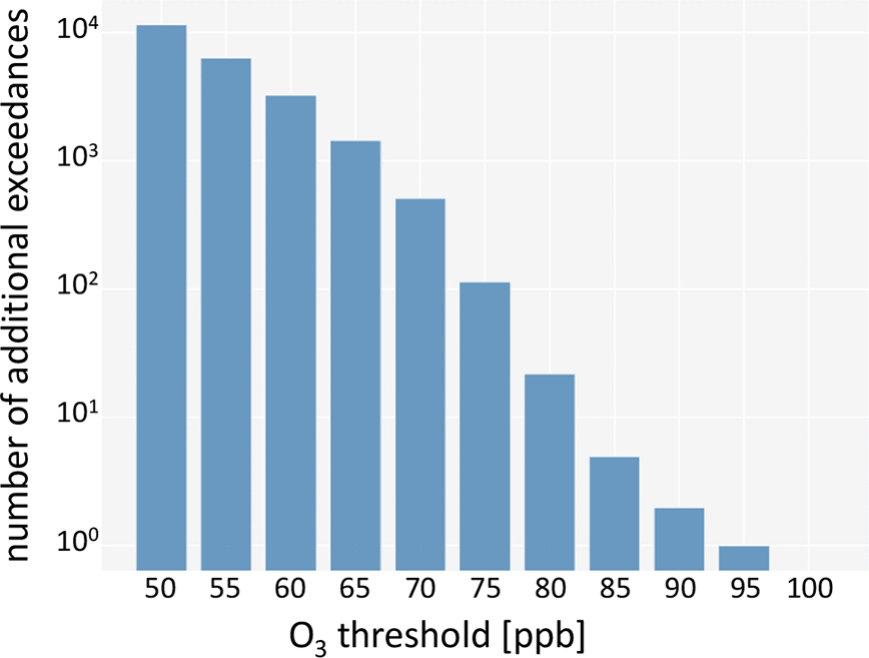
Number of additional exceedances of ozone thresholds contained in the data set after imputation of the data.

As mentioned in the Introduction, the number of exceedances of ozone concentration thresholds is an important indicator of the assessment of air quality. One example is the number of exceedances of daily maximum 8 h values greater than 70 ppb during the summer (nvgt70 summer).^22^ As a proof of concept, we count the number of additional threshold exceedances that the imputed data set contains (Figure 6). Of the total number of about 3.6 × 10^5^ imputed values, about 10^4^ samples yield ozone values above 50 ppb. Regarding the nvgt70 metric, 512 samples were imputed to the data set which exceed the threshold of 70 ppb. This shows that data imputation with our method can improve the robustness of air quality assessments.

**Figure 7:**
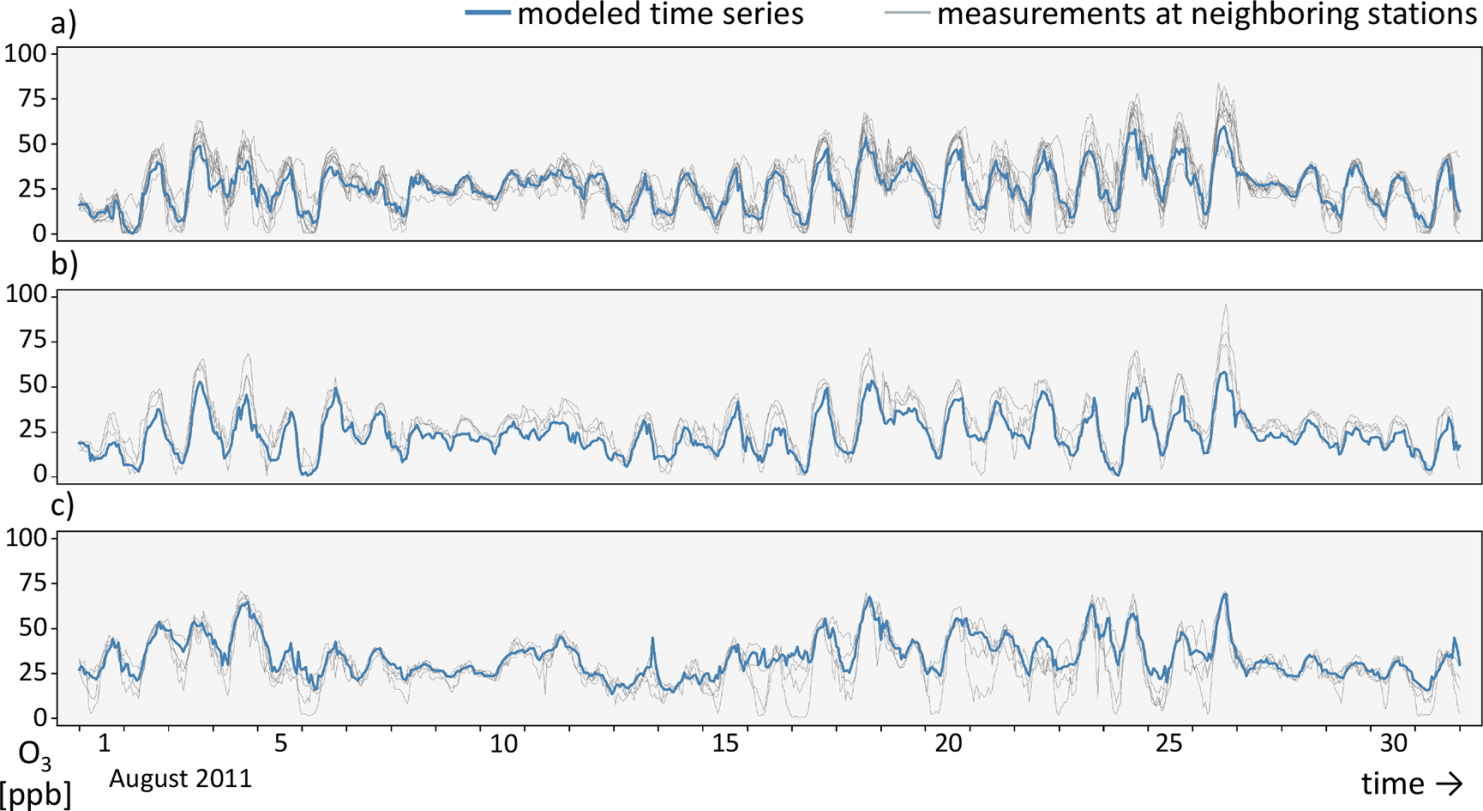
One-month-long excerpt of simulated ozone time series at three locations in Germany. They were modeled using our random forest and were correct and smooth. For comparison, the available measurements at stations within a radius of 50 km around the modeled locations are given. (a) Urban traffic location in the city of Borna, Sachsen, with 10 neighboring stations. (b) Urban traffic location in the city of Magdeburg with 4 neighboring stations. (c) Modeled time series in a rural background area west of Kassel with 5 neighboring stations.

As a second proof of concept, we imputed ozone data at station locations where no data were reported at all (Figure 7). We expect the evaluation metrics of these longer modeled ozone time series to be similar to longer gaps at single stations (Table 2), even though validation is impossible. The modeled time series is less variable than those measured at neighboring stations. This is because any (ozone) model, machine learning or otherwise, has problems predicting extremes.^67^ Dips and peaks in the measured time series can sometimes be attributed to noise due to short-term or small-scale effects on ozone that are not resolved in the auxiliary data and therefore are not represented in the model. Some of this is improved by correct and smooth: If all neighboring stations have a peak where the base model does not, then it is well corrected. An example can be seen in panel (c) of Figure 7 at time step 26.

## Discussion

### Imputing Missing Data in the UBA Data Set

The goal of this work was to impute missing ozone data at 278 stations of the UBA network in the year 2011. By fusing a variety of auxiliary data and available measurements using (graph) machine learning, high-accuracy imputations can be achieved. We applied other published methods as baseline methods to the UBA data set to compare our method with them. A direct comparison with the evaluation metrics reported in other studies may be misleading because they use different data sets, gap characteristics, and evaluation metrics than we do. We have chosen common baseline methods, namely, (1) imputation with mean values as is often implicitly done in the calculation of ozone metrics,^13,22^ (2) nearest-neighbor hybrid which is the best statistical method found by Junninen et al.,^23^ (3) EAC4 atmospheric reanalysis,^50^ and (4) random forest, which is a state-of-the-art machine learning method for structured data. Our method achieves equal or higher imputation accuracy than these other methods, depending on the gap characteristics. Also, our method is robust, with reasonable variations in the evaluation metrics given different numbers of neighbors, the iterative data splitting, and the length of the gaps.

Unlike approaches such as physics-guided machine learning,^68^ our method relies on the geospatial and statistical properties of the ozone data and auxiliary data without considering the physical or chemical processes mentioned in the Introduction. A strength of the correct and smooth method is that the correction step accounts for influences that the base models cannot predict without specifying those influences. Instead, it corrects the prediction by assuming that neighboring data points are subject to the same unknown influences; i.e., their base model errors are correlated. Smoothing ozone values across the graph structure defined on the monitoring network, as performed in the third step of correct and smooth, is a strongly simplified implementation of the ozone transport and diffusion processes. It does not consider wind speed or direction. Even though this works reasonably well, it should be improved in future models. Considering the spatiotemporal inhomogeneity of ozone and of air pollution in general, we have considered the local to regional differences in ozone levels by including both precursor emissions and meteorological parameters in our base models. We have furthermore used measurements of monitoring stations in the radius of 50 km around a missing value wherever available to better account for local to regional variances in the pollution.

The described method is suitable for near-real-time operational settings such as an imputation application for the TOAR data analysis services. Such a service is useful considering that the final validated UBA data set will not be available until the following year. Linear interpolation, random forest, and correct and smooth are comparably cheap algorithms that only take seconds to minutes to execute. Therefore, a near-real-time imputation of data can be potentially achieved by using these algorithms.

### Prospects for Graph Machine Learning in Air Quality Research

We showed that graph machine learning is suitable to be used with ozone data of the UBA monitoring network due to the irregular structure of the available data. We expect our findings to apply to other air quality data as well, although further studies would be needed to assess the imputation results for variables with different statistical properties, such as nitrogen oxide or particulate matter concentrations. One advantage of correct and smooth is that it can be used with any other feature-based method and for bias correction of numerical models.

With the definition of one data point as one node and the basing of the edge definition on the spatiotemporal distance between the nodes, the graph definition we used is relatively simple. More sophisticated approaches that should be explored in the future include time-resolved graphs^69^ for spatiotemporal machine learning or transformer architectures,^70^ which can learn to attend to the most helpful features in unstructured data. These architectures could be trained to take transport and advection of air pollutants into account by incorporating wind directions.^19^ One promising approach is also to infer the graph from the underlying data set.^47,71^ To further explore how the graph structure affects the results and what parameters are most crucial, sensitivity studies are necessary.

Many studies impute missing concentrations of multiple pollutants simultaneously and with varying input data available.^23,24^ From a graph perspective, this would require an algorithm that could handle different kinds of nodes with different kinds of labels. An algorithm like this would be especially interesting when real measurements of auxiliary data are used instead of reanalyses, because air quality measurements and measurements of meteorological parameters are often not reported from the same stations or they may have gaps themselves.

### Further Applications

The study presented here works on a spatially (Germany) and temporally (year 2011) limited domain. The only prerequisites to using this method in a different domain would be a similar spatial coverage of measurement stations and the availability of similar auxiliary data. Reasonably dense station networks exist in large parts of Europe, the United States, and East Asia but not in other world regions such as South America and Africa.^13^ Besides the lack of neighboring air quality stations, there may also be larger biases in the auxiliary data as documented, for example, with respect to the CAMS emissions and reanalyses.^50,51^

Besides missing data imputation, the method developed here could also be adapted for other questions posed in air quality research. One example is quality control—a common problem of graph theory is to flag untrustworthy nodes, such as untrustworthy Web sites or untrustworthy transactions.^72^ Similarly, untrustworthy measurements could be flagged with our method. This study showed that the method could predict meaningful ozone concentrations at places or time steps without measurements (Figure 7). Technically it would also be possible to predict the ozone time series at all grid points of a regular grid and therefore provide gridded ozone fields. This would be a logical extension of the mapping study by Betancourt et al.^48^

## Data Availability

The training data, including the graph data set, can be found under, if needed, 10.23728/b2share.59281340dd37485eb2c6a08de3587c13. The imputed data set can be found under 10.23728/b2share.04821864a81f40af89c7633889f147cb. The code which can be used to reproduce the experiments presented in this study can be found under https://gitlab.jsc.fz-juelich.de/esde/machine-learning/ozone-imputation.

## Supporting Information Available

More information on the ozone data used in this study and the hyperparameters for the correct and smooth methods (PDF)
