# Supplementary material for: Graph Machine Learning for Improved Imputation of Missing Tropospheric Ozone Data
Source: Environ Sci Technol. 2023 Sep 4;57(46):18246–58. doi: 10.1021/acs.est.3c05104 (PMC10666531; doi:10.1021/acs.est.3c05104)
Supplement: Supplementary file 1 — es3c05104_si_001.pdf [file es3c05104_si_001.pdf]

Graph machine learning for improved imputation of missing tropospheric ozone data

Supporting Information

Clara Betancourt, Cathy W. Y. Li, Felix Kleinert, and Martin G. Schultz

3 pages, 1 figure, 2 tables

S1: Basic ozone statistics

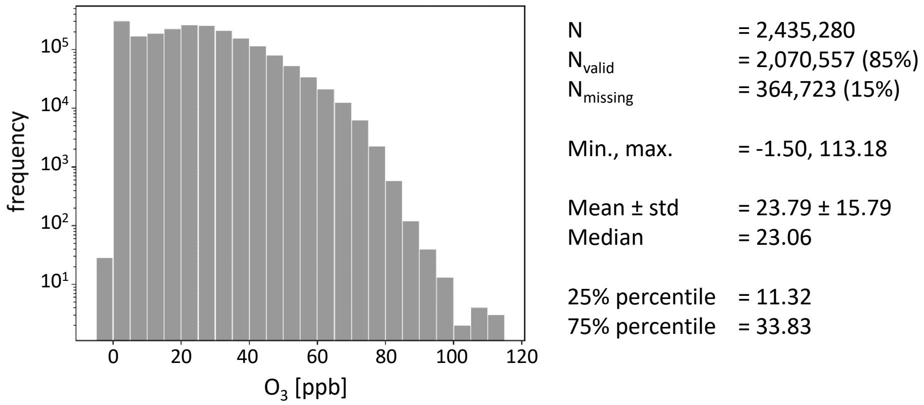

Figure S1: Histogram and summary statistics of the ozone data used in this study. N is the count, and all other statistics are given in parts per billion (ppb). Concentrations below zero may occur due to slight calibration errors.

S2: Gap lengths statistics

gap length and type	missing data n_{gaps}	$n_{data\ points}$	validation set n_{gaps}	$n_{data\ points}$	test set n_{gaps}	$n_{data\ points}$
single station						
1 h	42,103	42,103	21,052	21,052	21,052	21,052
2 h	4,226	8,452	2,113	4,226	2,113	4,226
3 - 5 h	2,536	9,399	1,267	4,624	1,267	4,611
6 - 23 h	3,226	52,575	1,612	24,536	1,612	24,485
1 - 6 d	386	21,136	186	8,772	186	8,800
$\geq$ 7 d	91	134,962	36	46,271	36	45,364
all 278 stations						
3 - 5 h	90	74,256	45	32,965	45	33,291
6 - 23 h	2	10,101	-	-	-	-
1 - 6 d	1	11,739	2	11,068	2	11,048
	$\sum$	364,723	$\sum$	153,514	$\sum$	152,877

Table S1: Gap lengths of the ozone data. We differ between missing data, validation set, and test set as described in the Data and Methods Section.

S3: Hyperparameters

	α_1	γ	α_2	$l_{1,2}$
search space	[0.0, 0.2, ..., 1.0]	[0.0, 0.25, ..., 2.5]	[0.0, 0.2, ..., 1.0]	[5, 10, ..., 20]
spatiotemporal mean	0.6,	2.0	0.0	10
spatial mean	0.6	1.75	0.2	10
nearest neighbor hybrid	0.0	0.0	0.2	10
EAC4 reanalyses	0.6	2.0	0.2	10
random forest	0.6	2.0	0.0	10

Table S2: Hyperparameters for correct and smooth in connection with the different base models.
